# Boosting capacitive performance of manganese oxide nanorods by decorating with three-dimensional crushed graphene

**DOI:** 10.1186/s40580-022-00300-2

**Published:** 2022-02-21

**Authors:** Akter Hossain Reaz, Shimul Saha, Chanchal Kumar Roy, Md Abdul Wahab, Geoffrey Will, Mohammed A. Amin, Yusuke Yamauchi, Shude Liu, Yusuf Valentino Kaneti, Md. Shahriar Hossain, Shakhawat H. Firoz

**Affiliations:** 1 Department of Chemistry, Bangladesh University of Engineering and Technology, Dhaka, 1000 Bangladesh; 2 Department of Chemistry, Jashore University of Science and Technology, Jashore, 7408 Bangladesh; 3 Australian Institute for Bioengineering and Nanotechnology (AIBN), The University of Queensland, Brisbane, QLD 4072 Australia; 4 School of Mechanical, Medical and Process Engineering, Faculty of Engineering, Queensland University of Technology, Brisbane, QLD 4000 Australia; 5 Department of Chemistry, College of Science, Taif University, P.O. Box 11099, Taif, 21944 Saudi Arabia; 6 JST-ERATO Yamauchi Materials Space-Tectonics Project and International Center for Materials Nanoarchitectonics (WPI-MANA), National Institute for Materials Science, Tsukuba, Ibaraki 305-0044 Japan; 7 School of Mechanical and Mining Engineering, Faculty of Engineering, Architecture, and Information Technology (EAIT), The University of Queensland, Brisbane, QLD 4072 Australia

**Keywords:** Supercapacitors, Reduced graphene oxide, Manganese oxide, Energy storage, Three-dimensional architecture

## Abstract

This work reports the rational design of MnO_x_ nanorods on 3D crushed reduced graphene oxide (MnO_x_/C-rGO) by chemical reduction of Ni-incorporated graphene oxide (GO) followed by chemical etching to remove Ni. The resulting MnO_x_/C-rGO composite synergistically integrates the electronic properties and geometry structure of MnO_x_ and 3D C-rGO. As a result, MnO_x_/C-rGO shows a significantly higher specific capacitance (*C*_sp_) of 863 F g^−1^ than MnO_x_/2D graphene sheets (MnO_x_/S-rGO) (373 F g^−1^) and MnO_x_ (200 F g^−1^) at a current density of 0.2 A g^−1^. Furthermore, when assembled into symmetric supercapacitors, the MnO_x_/C-rGO-based device delivers a higher *C*_sp_ (288 F g^−1^) than MnO_x_/S-rGO-based device (75 F g^−1^) at a current density of 0.3 A g^−1^. The superior capacitive performance of the MnO_x_/C-rGO-based symmetric device is attributed to the enlarged accessible surface, reduced lamellar stacking of graphene, and improved ionic transport provided by the 3D architecture of MnO_x_/C-rGO. In addition, the MnO_x_/C-rGO-based device exhibits an energy density of 23 Wh kg^−1^ at a power density of 113 Wkg^−1^, and long-term cycling stability, demonstrating its promising potential for practical application.

**Graphical Abstract:**

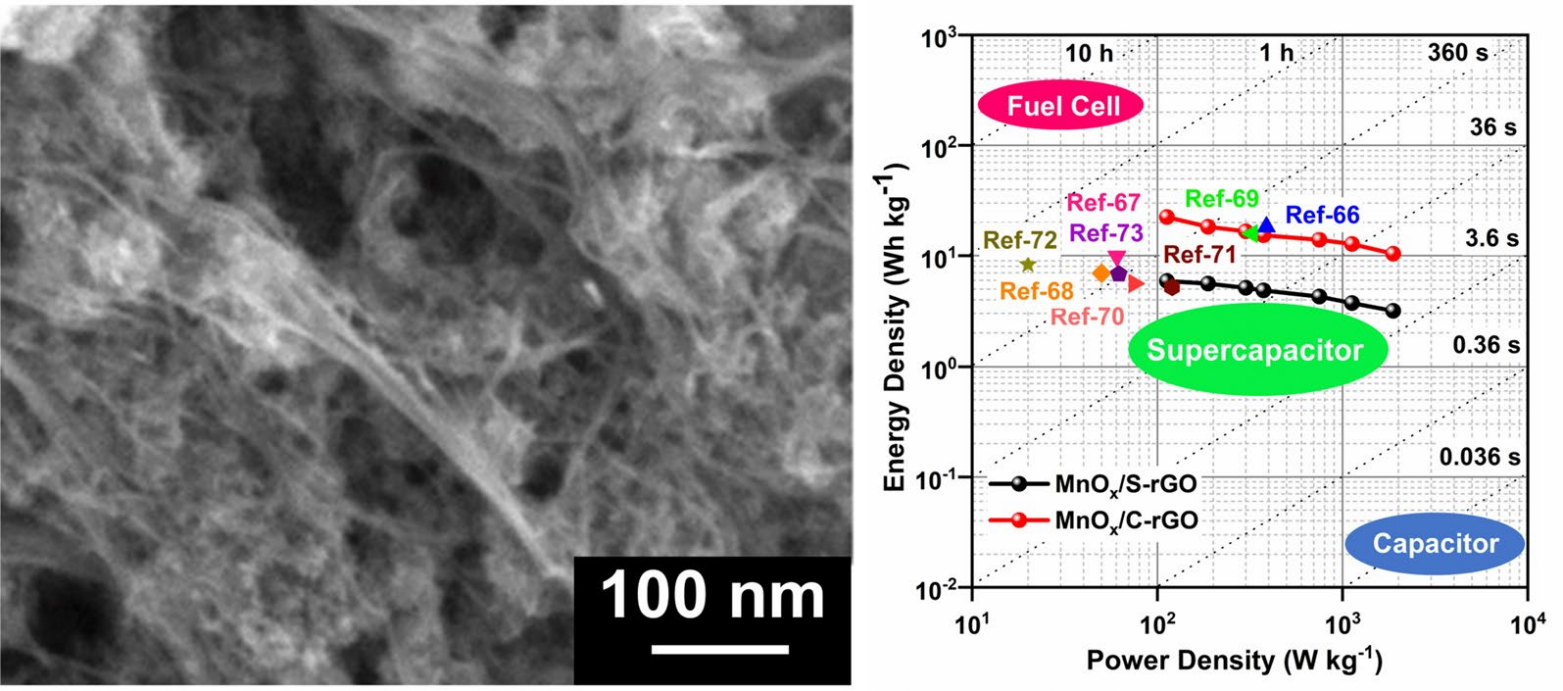

**Supplementary Information:**

The online version contains supplementary material available at 10.1186/s40580-022-00300-2.

## Introduction

Supercapacitors are promising energy storage devices owing to their fast charge/discharge rate, excellent cyclic stability, high power density, and good environmental compatibility [1–6]. However, extensive practical applications of supercapacitors require improving the performance of the electrode materials [7–11]. Among various transition-metal-based electrode materials, manganese dioxide (MnO_2_) has been considered as a potential electrode material for supercapacitors due to its high theoretical capacitance of 1370 F g^−1^ [12–14]. However, the practical capacitance of MnO_2_ is usually much lower than the theoretical capacitance due to its inherent poor electrical conductivity and slow ion transport. Moreover, the electrochemical dissolution of MnO_2_ during charge–discharge cycles degrades the cycling stability, thus limiting its practical applications [14–17].

To date, different strategies, such as nanostructuring, defect engineering, hybridization, and surface modification have been used to improve the electrochemical performance of MnO_2_-based electrodes [1, 18, 19]. Among them, the hybridization of MnO_2_ with carbon-based nanomaterials, such as graphene, carbon nanotubes, fullerenes, etc., has been employed to overcome the existing limitations of MnO_2_ [20, 21]. It is worth mentioning that a two-dimensional (2D) graphene nanostructure with a single carbon atom layer of *sp*^2^ graphitic configuration offers superior electrical conductivity upon hybridization with metal oxides [22–24]. However, the reported MnO_2_/2D graphene composites usually show rapid capacitance decay due to the low accessibility of electrolyte ions into the electrode materials [25–29]. 2D graphene sheets are soft and can form conformal contact with a surface or each other with the high aspect ratio of 2D morphology, which increases the overlapping field and decreases the reciprocal distance (*d*), resulting in an intense attraction [30]. Moreover, 2D graphene sheets often result in irreversible agglomeration due to strong π–π interactions and van der Waals force. The van der Waals force-induced strong attraction between parallel sheets of 2D graphene correlates with the overlapping field and the fourth power of the reciprocal distance of separation (1/*d*^4^) [30, 31]. The consequent result is lamellar stacking in the horizontal alignment of graphene microstructures, which hinders charge transport. Despite several approaches to resolve such agglomeration in 2D sheets (e.g., graphene sheets), the inherent drawbacks of 2D-graphene sheets (S-rGO) associated with the irreversible agglomeration and restacking of S-rGO have not been solved completely.

Recently, compositing MnO_2_ with 3D graphene instead of 2D graphene has been proposed as a potential way to prevent aggregation and lamellar stacking of rGO [30, 32–34]. Additionally, the large surface area and the fast and reversible Faradic redox reactions of 3D-structured materials can greatly improve electrochemical performance. 3D graphene/MnO_2_ composite has been successfully synthesized by combining chemical vapor deposition (CVD) and hydrothermal methods. This composite had a *C*_sp_ of 333 F g^−1^ at 0.2 A g^−1^ [33]. Biomass-derived N-doped 3D graphene@MnO_2_ (N-G@MnO_2_) composite was previously prepared by CVD method and it displayed a high *C*_sp_ of 411.5 F g^−1^ at 0.5 A g^−1^ [34]. However, both composites were fabricated using the CVD process which typically requires special apparatus and produces highly toxic gaseous as by-products [35]. Lin et al*.* [36] reported the synthesis of well-organized *ε*-MnO_2_ hollow spheres/rGO composites which exhibited a *C*_sp_ of 471 F g^−1^ at 0.8 A g^−1^. Although the use of 3D-structured composites of MnO_2_ and rGO may lead to a higher electrochemical performance, the fabrication of such composites is complicated and requires special attention to maintain the uniformity of the 3D structure.

Herein, we demonstrate a facile chemical route to fabricate MnO_x_ on 3D crushed rGO (MnO_x_/C-rGO). The 3D-crushed graphene structure enhances the surface-to-volume ratio, while also enabling a high accessible surface area for fast ion transportation. The large number of voids and junction defects introduced by the highly disordered 3D-crushed structure provides sufficient active sites to more ions. Furthermore, the network of MnO_x_ nanorods on the rGO matrix can help to reduce particle aggregation and prevent the restacking of rGO. As a result, the MnO_x_/C-rGO composite delivers a higher *C*_sp_ than both MnO_x_/S-rGO and MnO_x_. Moreover, the capacitive performance of the MnO_x_/C-rGO composite is also superior compared to MnO_x_/S-rGO.

## Experimental

### Materials

Graphite powder (< 20 µm with purity > 99.9 wt.%, Sigma Aldrich, Germany), sodium nitrate (NaNO_3_; Lab-Scan, Ireland), potassium permanganate (KMnO_4_; Sigma Aldrich, Germany), sulfuric acid (18.4 M H_2_SO_4_; Lab-Scan, Ireland), hydrogen peroxide (H_2_O_2_; Sigma Aldrich, Germany), hydrochloric acid (12 M HCl; Lab-Scan, Ireland), aqueous ammonia (NH_4_OH; Lab-Scan, Ireland), hydrazine hydrate (Sigma Aldrich, Germany), benzyl alcohol (Merck, Germany), ethanol (Lab-Scan, Ireland), nickel chloride hexahydrate (NiCl_2_.6H_2_O; Merck, Germany), sodium hydroxide (NaOH; Lab-Scan, Ireland), sodium borohydride (NaBH_4_; Sigma Aldrich, Germany), ethylene glycol (Sigma Aldrich, Germany), polyvinylidene fluoride (PVDF; Sigma Aldrich, Germany), *N*-methyl-2-pyrrolidone (NMP; Sigma Aldrich, Germany) and sodium sulfate (Na_2_SO_4_; Sigma-Aldrich, Germany) were purchased and used without further purification. A water purification system (Barnstead nano pure, Thermo Scientific, USA) was used to obtain deionized (DI) water. A digital ultrasonic bath (Powersonic 505, Hwashin, South Korea) was used for sonication.

### Preparation of MnO_x_/S-rGO and MnO_x_/C-rGO

Graphene oxide (GO) was prepared from graphite powder following the modified Hummers method [37, 38]. A mixture of graphite powder (1.0 g), NaNO_3_ (0.5 g), KMnO_4_ (3.0 g), and DI water (46 mL) was prepared. A concentrated H_2_SO_4_ solution (23 mL) was slowly added into the mixture under strong stirring at about 35 °C. The temperature of the mixture was raised to about 98 °C and kept for 15 min. After that, the temperature of the mixture was lowered to 60 °C with the addition of 140 mL of warm DI water and 10 mL H_2_O_2_ (30% *w*/*v*). The mixture was further stirred for 2 h under continuous stirring to obtain the GO suspension. This suspension was washed several times with 1 M HCl solution. DI water was used to completely remove chloride ions. The suspension was dried in an oven to obtain GO powder. To synthesize rGO, a GO suspension was prepared by adding 360 mg of the GO powder into 400 mL of DI followed by ultrasonication. Aqueous ammonia (10 mL, 25% *w*/*v*) and hydrazine hydrate (10 mL, 80%) were then added under continuous stirring for 2 h at 98 °C. The dispersed 2D rGO sheets (S-rGO) were filtrated and dried in a drying oven at 60 °C. The synthesized S-rGO was used to prepare MnO_x_/S-rGO by mixing benzyl alcohol (100 µL) with the prepared S-rGO (200 mg) and DI water (30 mL) under slow stirring followed by the addition of KMnO_4_ (60 mg). The mixture was then stirred for 10 h at 60 °C. The obtained black MnO_x_/S-rGO powder was washed with DI water and ethanol. The solid powder was dried at 50 °C for 12 h in a drying oven and stored for further use.

The C-rGO sample was obtained by first preparing Ni@rGO core–shell, where the Ni nanoparticles were synthesized according to a previous report [39]. Typically, 1000 mg of Ni NPs was dispersed in 100 mL ethylene glycol solution and sonicated for 60 min to obtain EG-wrapped Ni NPs. The previously prepared GO was then mixed with the EG-wrapped Ni NPs at a weight ratio of 1:5. The mixture was sonicated for 1 h followed by 24 h stirring, which resulted in a blackish-brown gel. The mixture of hydrazine hydrate (5 mL; 80% *w*/*v*) and aqueous ammonia (5 mL; 25% *w*/*v*) was added into the resulting blackish-brown gel under continuous stirring for 2 h at 98 °C. The solid black mass of Ni@rGO was separated by centrifugation and washed several times with DI water. 3D C-rGO was obtained from the prepared Ni@rGO by leaching the Ni core using HCl (25 mL, 37%) solution. DI water and ethanol were used to wash the prepared product, which was collected by centrifugation and dried overnight in an oven at 60 °C. The MnO_x_/C-rGO was prepared using the same procedures as MnO_x_/S-rGO, except that C-rGO was used instead of S-rGO.

### Characterization

The surface morphology of the prepared composites was analyzed using field-emission scanning electron microscope (FESEM; JSM-7600F, JEOL, Japan) and transmission electron microscope (TEM, G2 F20, Tecnai, Japan). The phase composition and crystallinity of the samples were investigated using X-Ray diffractometer (XRD, Empyrean, PANalytical-Netherlands) equipped with Cu-K*α* radiation (*λ* = 1.5418 Å). X-ray photoelectron spectrometer (XPS, Kratos AXIS Nova, Kyoto, Japan) equipped with a monochromator Al Kα (*hv* = 1486.6 eV) was used to analyze the surface compositions of the samples.

### Electrochemical measurements

The electrochemical measurements were performed by cyclic voltammetry (CV), galvanostatic charge–discharge (GCD), and electrochemical impedance spectroscopy (EIS) using an electrochemical workstation, (CHI 660E, CH Instruments, USA). In the three-electrode system, a single compartment electrochemical cell was produced with the sample-coated graphite rod (99.9% pure; OtoolWorld, USA; surface area ~ 0.28 cm^2^) as the working electrode, platinum (Pt) wire as the counter electrode and Ag/AgCl (sat. KCl) as the reference electrode. The improved solvent casting and drop drying processes were used to prepare the working electrode. A homogeneous slurry of the coating material was prepared by mixing the active material (95%) and PVDF binder (5%) with 150 μL of NMP followed by ultrasonication for 1 h. The mirror-polished graphite rod was then coated with 15–20 μL of the prepared slurry and dried at 60 °C in a vacuum oven for 5 h. The areal active mass density was ~ 3 mg cm^−2^. The CV measurements were conducted at various scan rates from 5 to 100 mV s^−1^. The GCD tests were performed at different current densities from 0.2 to 5.0 Ag^−1^ using 0.5 M Na_2_SO_4_ as the electrolyte within a stable potential window of − 0.1 to 0.8 V.

For the two-electrode system, a customized coin cell compartment with disk-shaped graphite electrodes (surface area ~ 0.45 cm^2^) was used to evaluate the electrochemical performance of the prepared composites. The disk-shaped graphite electrodes were drop-casted by following the same procedures as described for the three-electrode system. Whatman filter paper soaked with aqueous 1.0 M Na_2_SO_4_ electrolyte was used as a separator and placed between two modified disk-shaped electrodes. Here, the CV measurements were performed at different scan rates from 5 to 100 mV s^−1^. The GCD experiments were performed at different current densities from 0.3 to 5.0 A g^−1^ using an aqueous 1.0 M Na_2_SO_4_ electrolyte within a potential range of 0 to 1.5 V. EIS measurements were conducted using the two-electrode system within a frequency range of 0.01 to 100,000 Hz at the open-circuit potential (OCP) with an AC amplitude of 10 mV at room temperature. The long-term cyclability of the prepared electrode materials was performed by repeating GCD cycles up to 10,000 times at a current density of 5 A g^−1^ and potential range of 0 to 1.5 V in the two-electrode system. The voltage holding test was performed by subjecting a maximum potential of 1.5 V for 60 h after charging at a current density of 2 A g^−1^.

In the three-electrode system, the specific capacitance (*C*_sp_) was calculated from the GCD curves using Eq. (1) [29]

1 $$C_\mathrm{sp }(\mathrm{F g}^{-1}) =\frac{\mathrm{\it I\Delta \it t}}{\mathrm{\it m\Delta \it V}}$$

where *I* is the loaded current, Δ*t* is the discharge time, Δ*V* is the potential range, and *m* is the mass loading of the active material. For the two-electrode system, *C*_sp_, energy density (*E*), and power density (*P*) were calculated using Eqs. (2)–(4) [40, 41].

2 $$C_\mathrm{sp }(\mathrm{F g}^{-1}) =\frac{2\mathrm{\it I\Delta \it  t}}{\mathrm{\it  m\Delta \it  V}}$$

3 $$E \,(\mathrm{Whkg}^{-1}) =\frac{{C}_{\mathrm{sp}}{\left(\mathrm{\Delta \it V}\right)}^{2}}{2\times 4\times 3600}$$

4 $$P\, (\mathrm{Wkg}^{-1}) =\frac{{E}_{d}\times 3600}{\mathrm{\Delta \it t}}$$

The Coulombic efficiency (*η*) was calculated using Eq. (5) [40, 41].

5 $$\eta =\frac{{\Delta t}_{discharge}}{\Delta {t}_{charging}}$$

where $\Delta$ *t* is the time duration of charge or discharge part of GCD cycles. The capacitance retention (CR) values were calculated using Eq. (6).

6 $$\mathrm{Capacitance\,retention } \mathrm{( CR)}=\frac{Final\,Capacitance}{Initial\,Capacitance}\times 100\mathrm{\%}$$

## Results and discussion

**Fig. 1:**
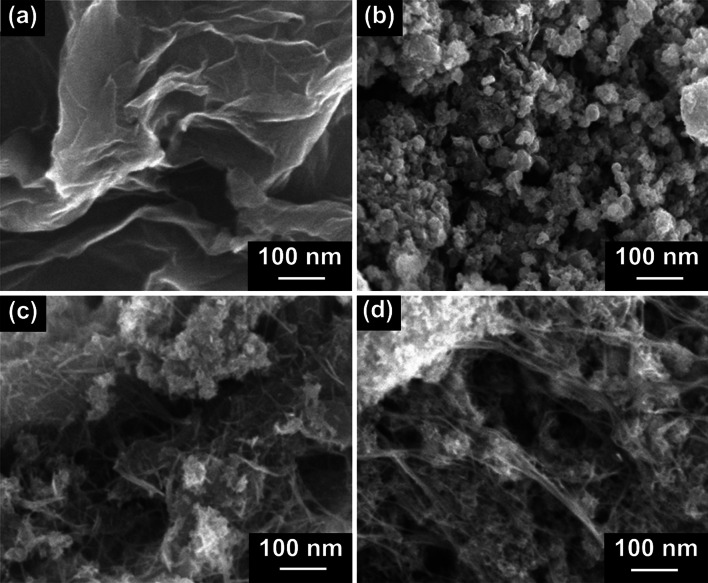
FESEM images of **a** S-rGO, **b** C-rGO, **c** MnO_x_/S-rGO, and **d** MnO_x_/C-rGO

The morphological analysis of the as-prepared samples was conducted by FESEM. The FESEM images of S-rGO, C-rGO, MnO_x_/S-rGO_,_ and MnO_x_/C-rGO are presented in Fig. 1. The S-rGO sample exhibits a flake-like architecture with isolated layers arranged intermittently edge-to-edge (Fig. 1a). The nanoscale interlocking of graphene sheets is responsible for this structure [42, 43]. The FESEM image of C-rGO is displayed in Fig. 1b. The C-rGO sample shows a nanoball-like structure with a crushed feature. The crushed graphene is formed due to the instability of graphene nanoballs as a result of the void formed at the core of Ni@rGO by etching with HCl solution. Figure 1c depicts the surface morphology of the MnO_x_/S-rGO composite. An interconnected network-like composite is formed with MnO_x_ which covers the sheet-like S-rGO. However, these graphene nanosheets are slightly disrupted and not well-stacked. It is assumed that during the assembly of MnO_x_ nanorods on the surface of the graphene nanosheets, a disruptive force separates these graphene sheets.

**Fig. 2:**
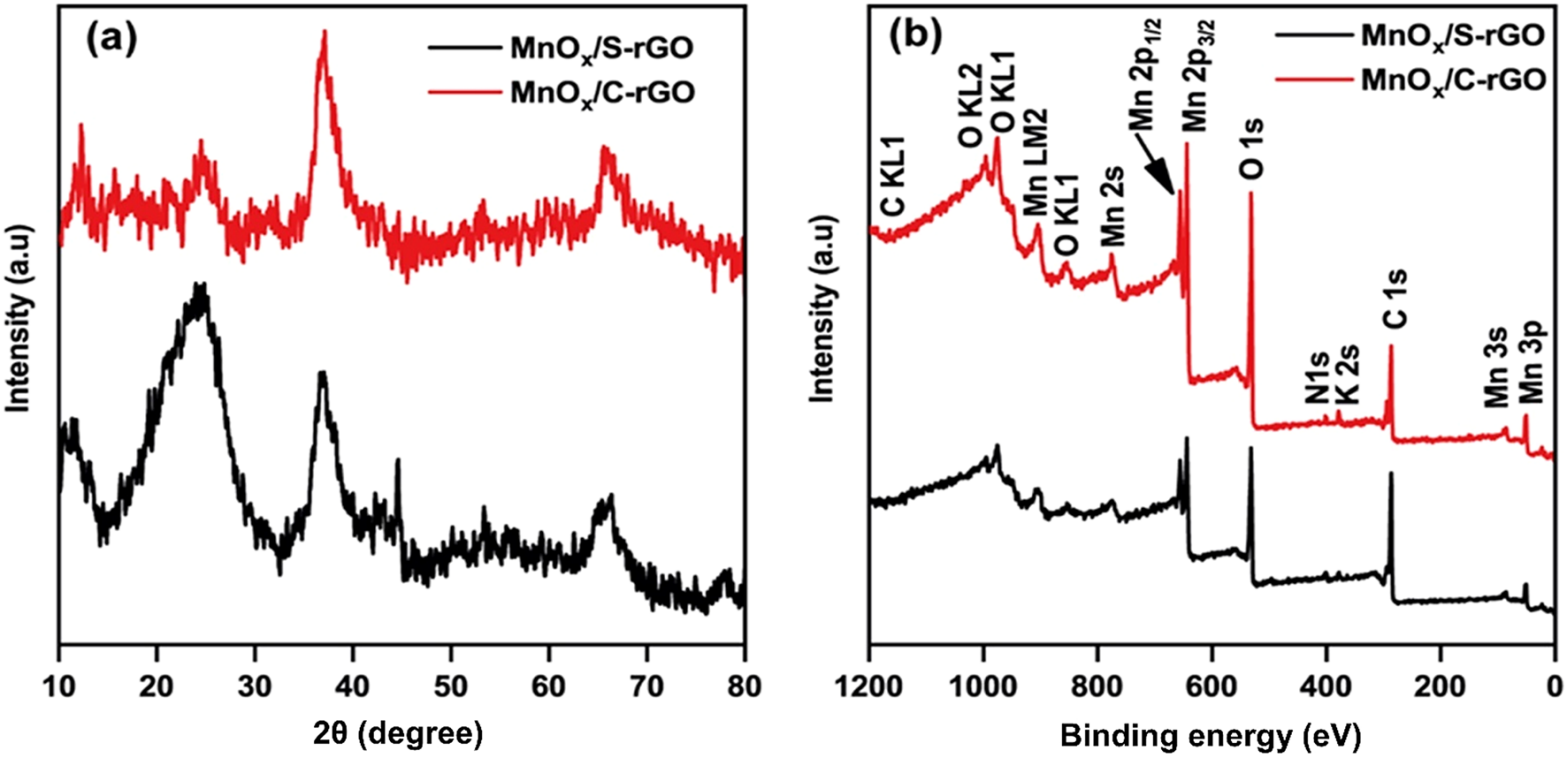
**a** XRD patterns and **b** XPS survey spectra of MnO_x_/S-rGO and MnO_x_/C-rGO

Figure 1d illustrates the FESEM image of the MnO_x_/C-rGO composite, in which many MnO_x_ nanorods cover the C-rGO surface with high homogeneity. Interestingly, the ball-like shape of C-rGO is absent in the MnO_x_/C-rGO composite. The C-rGO is crushed and distributed in a well-dispersed network of MnO_x_ nanorods. The TEM images (Additional file 1: Fig. S1a and b) show the formation of nanorod-shaped MnO_x_ on S-rGO in MnO_x_/S-rGO and C-rGO in MnO_x_/C-rGO. These images also confirm the formation of layered birnessite MnO_x_. XRD was used to explore the crystal structures of the as-prepared samples. XRD patterns of MnO_x_/S-rGO and MnO_x_/C-rGO are shown in Fig. 2a. The diffraction peak at 2*θ* = 25° can be assigned to the (002) plane of rGO in MnO_x_/S-rGO and MnO_x_/C-rGO.[17] Here, the (002) peak in MnO_x_/S-rGO is broader than that of MnO_x_/C-rGO, indicating the more amorphous nature of S-rGO in MnO_x_/S-rGO. The diffraction peaks at 11.6°, 24.5°, 36.6° and 66° in the XRD patterns of MnO_x_/S-rGO and MnO_x_/C-rGO suggest the formation of a layered birnessite-type MnO_2_ phase (JCPDS No. 43-1456). The sharp peak at 36.6° corresponds to the ordered MnO_x_ nanorods present in MnO_x_/C-rGO.

XPS analysis was conducted to further investigate the surface chemistry of the prepared samples. The comparative XPS survey spectra of the MnO_x_/S-rGO and MnO_x_/C-rGO are shown in Fig. 2b. The C 1 s peak at 284.5 eV and the O 1 s peak at 532.0 eV indicate the presence of carbon and oxygen from graphene sheets. The deconvoluted O 1 s peak at 530.0 eV can be assigned to the oxygen bound to manganese (Mn−O) in MnO_x_ (Additional file 1: Fig. S2). The O 1 s peaks in both MnO_x_/S-rGO and MnO_x_/C-rGO composites at binding energies of 529.6, 530.0, and 532.4 eV are assigned to Mn–O–Mn, Mn–O–C, and C−O−C/C–OH bonds, respectively (Additional file 1: Fig. S2) [44, 45]. The above results confirm the formation of MnO_x_ on the graphene oxide surface. The O 1 s peaks at 532.4 and 533.48 eV correspond to C–O–H and C–O–C of rGO in MnO_x_/S-rGO and MnO_x_/C-rGO composites, respectively (Additional file 1: Fig. S2).[46, 47] The deconvoluted C 1 s peaks of rGO reveal the presence of non-oxygenated carbon (C=C/C–C) at 284.6 eV, the epoxy group at 285.2 eV, and carbonyl carbon (C=O/–COOH) at 288.4 eV (Additional file 1: Fig. S3) [47–49]. The presence of N-doped graphene (NG) is also observed, as indicated by the small peak for N 1 s at ~ 400 eV in the XPS spectra [45]. The Mn 2p XPS spectra exhibit two characteristic peaks at 642.0 and 652.5 eV (Fig. 2b), corresponding to Mn 2p_3/2_ and Mn 2p_1/2_ spin–orbit peaks of MnO_x_, respectively, further confirming the presence of MnO_x_ in the composite. The deconvoluted Mn 2p_3/2_ XPS spectra (Additional file 1: Fig. S4) of the MnO_x_/S-rGO and MnO_x_/C-rGO composites reveal the presence of two peaks centered at 642 and 644 eV corresponding to the spin–orbit doublet of Mn 2p_3/2_, confirming the presence of both Mn^3+^ and Mn^4+^ in these composites. The XPS analysis suggests the existence of Mn_2_O_3_ and MnO_2_ in the prepared composites, which is thus defined as MnO_x_ in this study.[44, 49, 50] A small contribution of Mn(VII) from potassium manganate is confirmed by the presence of a trace amount of potassium on the surface in MnO_x_/S-rGO_._ It is noted that there is much more potassium in the MnO_x_/C-rGO composite without much increase of the Mn species. This K^+^ has a vital role in forming the layered birnessite phase. Some Mn^4+^ ions at the center of the MnO_6_ octahedral are replaced by Mn^3+^ ions, generating a net negative charge. The cations compensate for the net negative charges, leading to the formation of birnessite phase [25, 51–53].

**Fig. 3:**
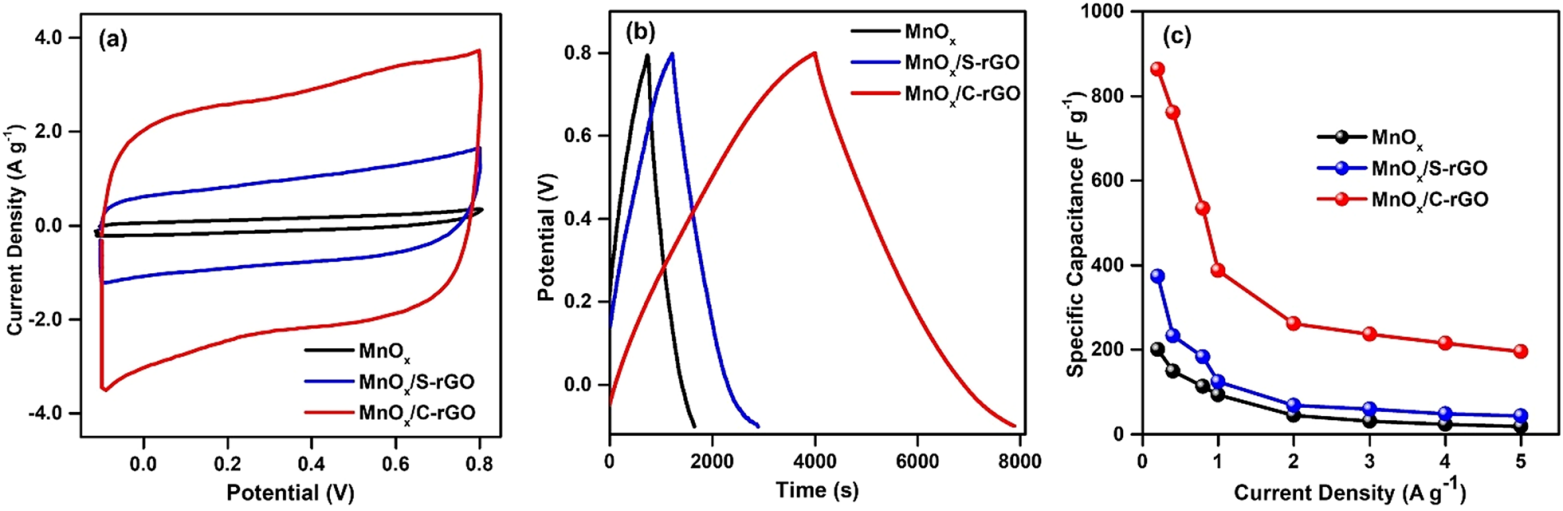
Three-electrode system measurements: **a** CV curves at a scan rate of 50 mV s^−1^; **b** GCD curves at a current density of 0.2 Ag^−1^, and **c** specific capacitances at different current densities for MnO_x_, MnO_x_/S-rGO, and MnO_x_/C-rGO electrodes

Figure 3a shows the comparative CV curves of MnO_x_/S-rGO and MnO_x_/C-rGO obtained using a three-electrode system at a scan rate of 50 mV s^−1^ in the potential range of − 0.1 to 0.8 V in a 0.5 M Na_2_SO_4_ aqueous electrolyte. Both MnO_x_/S-rGO and MnO_x_/C-rGO composites exhibit symmetric rectangular CV curves, which are characteristic of EDLC. However, the integrated area of the CV curve of MnO_x_/C-rGO is significantly higher than that of MnO_x_/S-rGO. This indicates the higher charge storage capability of MnO_x_/C-rGO compared to MnO_x_/S-rGO [54–56]. The rectangular shape of the CV curves of MnO_x_, MnO_x_/S-rGO and MnO_x_/C-rGO is retained at all scan rates (5, 10, 20, 50, and 100 mV s^−1^), as shown in Additional file 1: Figs. S5a, S6a and S7a, respectively. The rectangular shape is well-retained with the increase of scan rate, indicating the good reversibility of the MnO_x_/C-rGO electrode.

Figure 3b displays the comparative GCD curves of the MnO_x_/S-rGO and MnO_x_/C-rGO composites at a current density of 0.2 A g^−1^. The MnO_x_/C-rGO composite exhibits a longer discharge time than MnO_x_/S-rGO. The GCD curves of MnO_x_, MnO_x/_S-rGO and MnO_x_/C-rGO at current densities; 0.2, 0.4, 0.8, 1, 2, 3, 4, and 5 A g^−1^ are shown in Additional file 1: Figs. S5b, S6b and S7b, respectively. The *C*_sp_ values of MnO_x_/S-rGO and MnO_x_/C-rGO were calculated from the discharge curves of GCD using Eq. (1). MnO_x_/C-rGO exhibits a higher *C*_sp_ (863 F g^−1^) than both MnO_x_/S-rGO (373 F g^−1^) and MnO_x_ (200 F g^−1^) at 0.2 A g^−1^. The *C*_sp_ values of MnO_x_/S-rGO and MnO_x_/C-rGO were also calculated individually at different current densities, as shown in Fig. 3c. The *C*_sp_ values of MnO_x_/C-rGO, MnO_x_/S-rGO, and MnO_x_ at a high current density of 5 A g^−1^ are 196, 43.0, and 20.0 F g^−1^, respectively. From Table 1, it can be observed that the prepared MnO_x_/C-rGO composite shows higher* C*_sp_ than previously reported MnO_2_-based composites [33, 57–63].

**Table 1:** Comparison of the specific capacitance of MnO_x_/C-rGO electrode against previously reported electrode materials for supercapacitors

Electrode	Electrolyte	Specific capacitance	References
MnO_x_/C-rGO	0.5 M Na_2_SO_4_	863 F g^−1^ at 0.2 A g^−1^	This work
MnO_2_/PC-Cs/MnO_2_	1.0 M KOH	397 F g^−1^ at 1 A g^−1^	[57]
MnO_2_/carbon/Ag	3.0 M KOH	628 F g^−1^ at 1 A g^−1^	[58]
MnO_2_/porous carbon	1.0 M Na_2_SO_4_	140 F g^−1^ at 0.3 A g^−1^	[59]
3D-graphene/MnO_2_	1.0 M Na_2_SO_4_	333 F g^−1^ at 0.2 A g^−1^	[33]
MnO_2_/graphene-like porous carbon	1.0 M Na_2_SO_4_	438 F g^−1^ at 0.5 A g^−1^	[60]
MnO_2_/reduced graphene oxide	6.0 M KOH	343 F g^−1^ at 0.5 A g^−1^	[61]
MnO_2_/rGO composite	1.0 M Na_2_SO_4_	194 F g^−1^ at 0.2 A g^−1^	[62]
MnO_2_/3D-carbon nanotubes-graphene	1.0 M Na_2_SO_4_	365 F g^−1^ at 1 A g^−1^	[63]

**Fig. 4:**
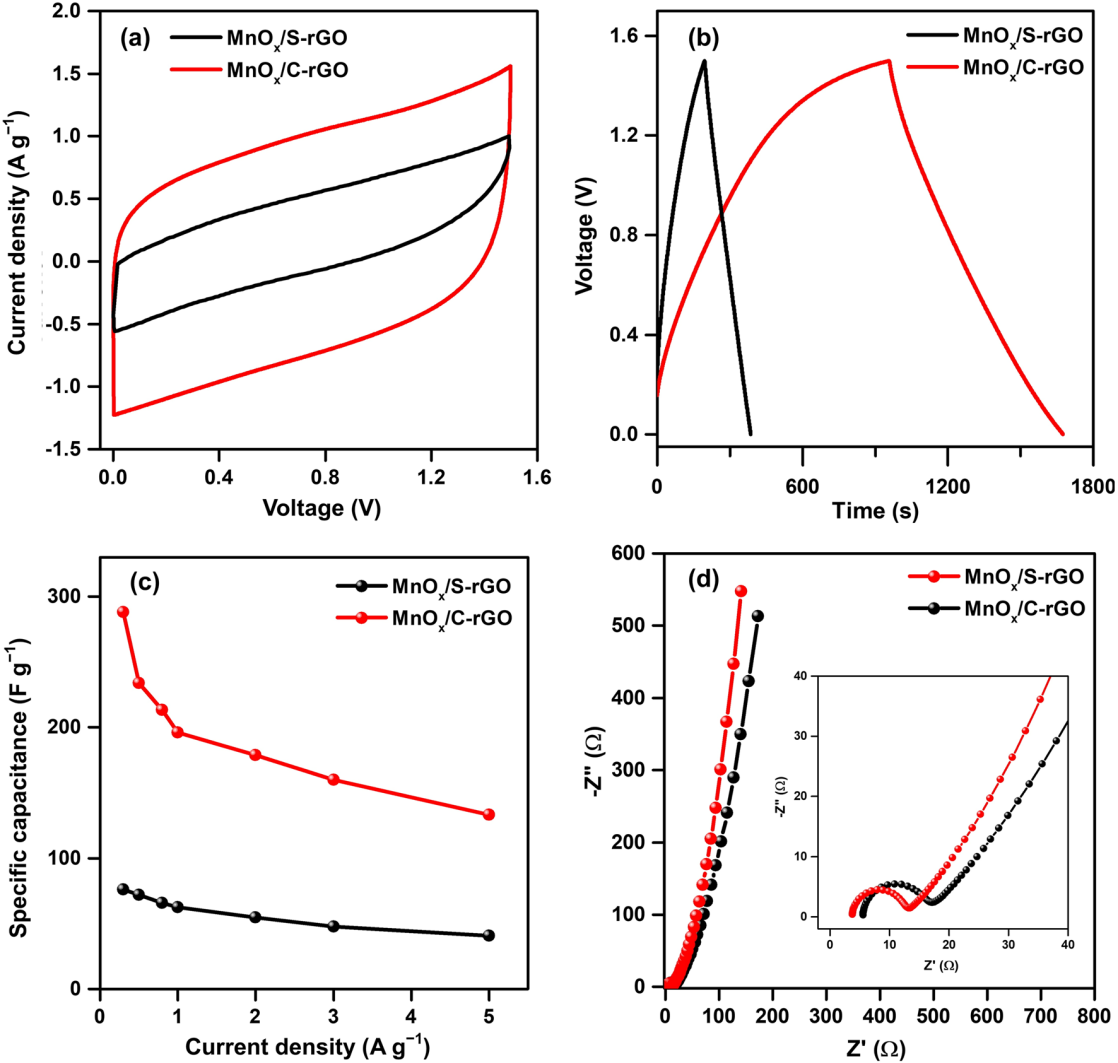
Two-electrode system measurements: **a** CV curves at a scan rate of 20 mV s^−1^; **b** GCD curves at a current density of 0.3 A g^−1^; **c** Specific capacitances of symmetric devices based on MnO_x_/S-rGO and MnO_x_/C-rGO at different current densities, and **d** Nyquist plots of symmetric devices based on MnO_x_/S-rGO and MnO_x_/C-rGO

Furthermore, symmetrical two-electrode supercapacitor devices with MnO_x_/S-rGO and MnO_x_/C-rGO were fabricated with a Whatman filter paper separator soaked in a 1 M Na_2_SO_4_ electrolyte. Figure 4a displays the comparative CV curves of the MnO_x_/S-rGO and MnO_x_/C-rGO-based symmetric devices at a scan rate of 20 mV s^−1^ in a potential range of 0 to 1.5 V. The symmetrical rectangular shape is present in the CV curves of both samples in the two-electrode system as well, indicating the EDLC behavior of both devices. With increasing scan rate, the MnO_x_/C-rGO-based device can maintain its CV curves, indicating its good rate performance (Additional file 1: Fig. S8a, b). The GCD curves of the MnO_x_/S-rGO and MnO_x_/C-rGO-based symmetric devices at a current density of 0.3 A g^−1^ are compared in Fig. 4b. The GCD curve of the MnO_x_/C-rGO-based device displays an almost symmetrical triangular shape, confirming its EDLC behavior. Moreover, the discharging time of the MnO_x_/C-rGO-based device is longer than that of the MnO_x_/S-rGO-based device. The GCD curves of the MnO_x_/S-rGO and MnO_x_/C-rGO-based symmetric devices at various current densities from 0.3 to 5 A g^−1^ are shown in Additional file 1: Fig. S8c, d, respectively. The *C*_sp_ values of the MnO_x_/S-rGO and MnO_x_/C-rGO-based devices at different current densities were calculated using Eq. (2).

The *C*_sp_ as a function of current density is plotted in Fig. 4c. The *C*_sp_ values of the MnO_x_/C-rGO-based symmetric device are 288, 234, 213, 196, 179, 160, and 133 F g^−1^ at current densities of 0.3, 0.5, 0.8, 1, 2, 3, and 5 A g^−1^, respectively. For comparison, the *C*_sp_ values of the MnO_x_/S-rGO-based symmetric device are 76, 72, 66, 62, 54, 47, and 40 at current densities of 0.3, 0.5, 0.8, 1, 2, 3, and 5 A g^−1^, respectively. Obviously, the *C*_sp_ values of the MnO_x_/C-rGO-based device are much higher than those of the MnO_x_/S-rGO-based device. The significantly enhanced capacitance of MnO_x_/C-rGO-based device may be attributed to the nanonet-like structure formed by the crushed rGO and MnO_x_ nanorods, which may provide an effective network for ion transport. EIS measurements were performed to analyze the charge transfer resistance in the MnO_x_/S-rGO and MnO_x_/C-rGO-based symmetric devices (Fig. 4d). The corresponding fitted equivalent circuits and the obtained values are presented in Additional file 1: Fig. S9 and Table S1, respectively. The *R*_S_ value of the MnO_x_/C-rGO-based device (3.742 Ω) is lower than that of the MnO_x_/S-rGO-based device (*R*_S_ = 5.531 Ω), suggesting the enhanced electrical contact between MnO_x_/C-rGO and electrolyte. The charge transfer resistance (*R*_CT_) value of the MnO_x_/C-rGO-based device (8.734 Ω) is lower than that of the MnO_x_/S-rGO-based device (10.454 Ω). The superior ideal capacitive behavior of the MnO_x_/C-rGO-based device is indicated by the higher verticality of its Nyquist plot in the low-frequency region compared to the MnO_x_/S-rGO-based device. The sharp edges of C-rGO with the random distribution of multi-nanostructure provide pores and free volume inside and between rGO layers, thus facilitating a multi-channel structure for ion diffusion in MnO_x_/C-rGO.

**Fig. 5:**
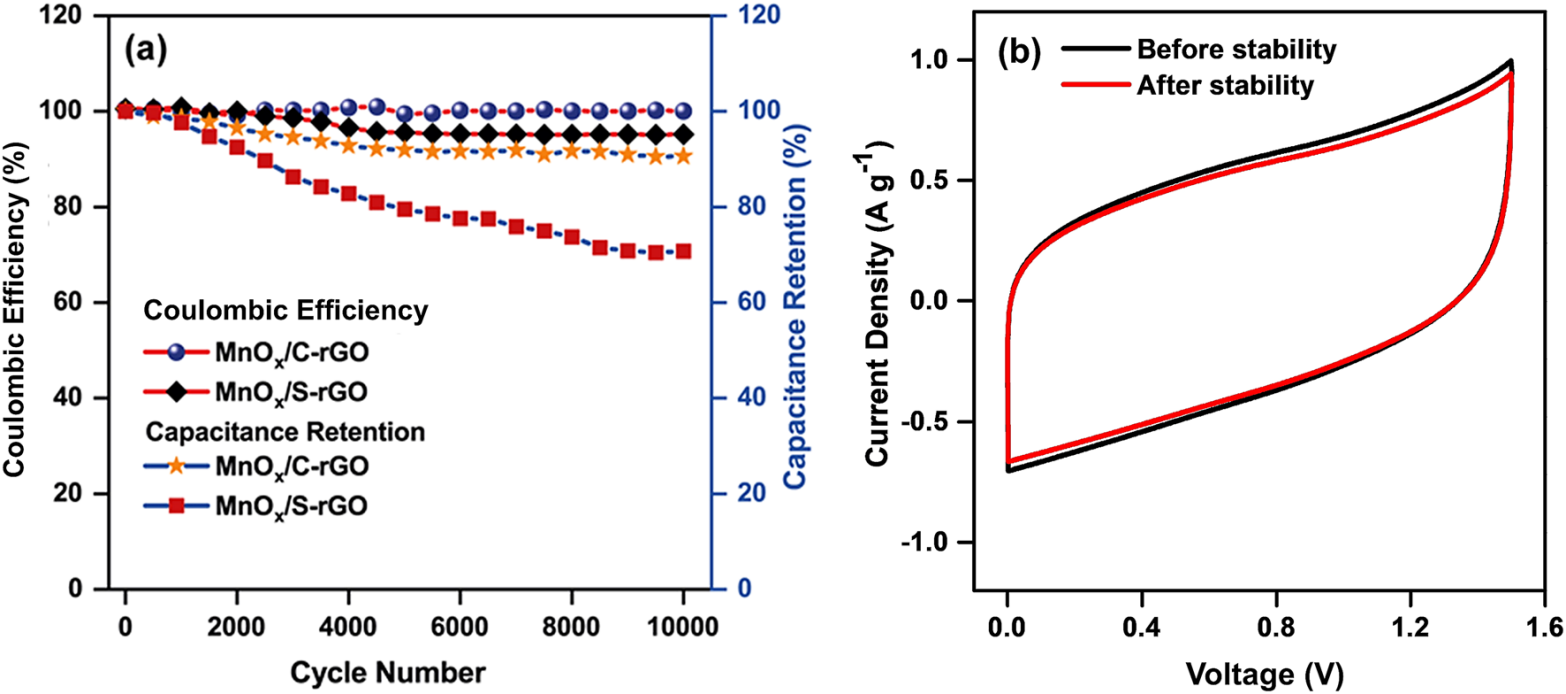
**a** Cycling stability of MnO_x_/S-rGO and MnO_x_/C-rGO-based symmetric devices. **b** CV curve of the MnO_x_/C-rGO-based device before and after stability test at 10 mV s^−1^ in 1.0 M Na_2_SO_4_

The cycling performance of the fabricated symmetric devices with MnO_x_/S-rGO and MnO_x_/C-rGO was performed in the voltage range of 0 to 1.5 V at 5 A g^−1^. The Coulombic efficiency was calculated using Eq. (5) and the capacitance retention was determined using Eq. (6). Figure 5a compares the Coulombic efficiency and capacitance retention of the symmetric devices with MnO_x_/S-rGO and MnO_x_/C-rGO over 10,000 GCD cycles. Approximately 100% Coulombic efficiency is achieved for the MnO_x_/C-rGO-based device over the total cycle. However, the symmetric device fabricated using MnO_x_/S-rGO shows a decrease in Coulombic efficiency with consequent GCD cycles. The MnO_x_/C-rGO-based device retains 90% of its initial capacitance after 10,000 GCD cycles, while the MnO_x_/S-rGO-based device fades rapidly with only 70% of its initial capacitance being retained. This indicates the superior cycling stability of the MnO_x_/C-rGO-based device relative to the MnO_x_/S-rGO-based device. The slight decrease in the capacitance retention of the MnO_x_/C-rGO-based device may be due to the dissolution and detachment of electrolyte or oxygen evolution during charge–discharge cycles [15, 64, 65]. However, the dissolution of manganese oxide is not excluded here [14, 15, 64, 65]. The CV curves of the MnO_x_/C-rGO-based device before and after the stability test are almost similar (Fig. 5b), thus confirming the good stability of this composite.

**Fig. 6:**
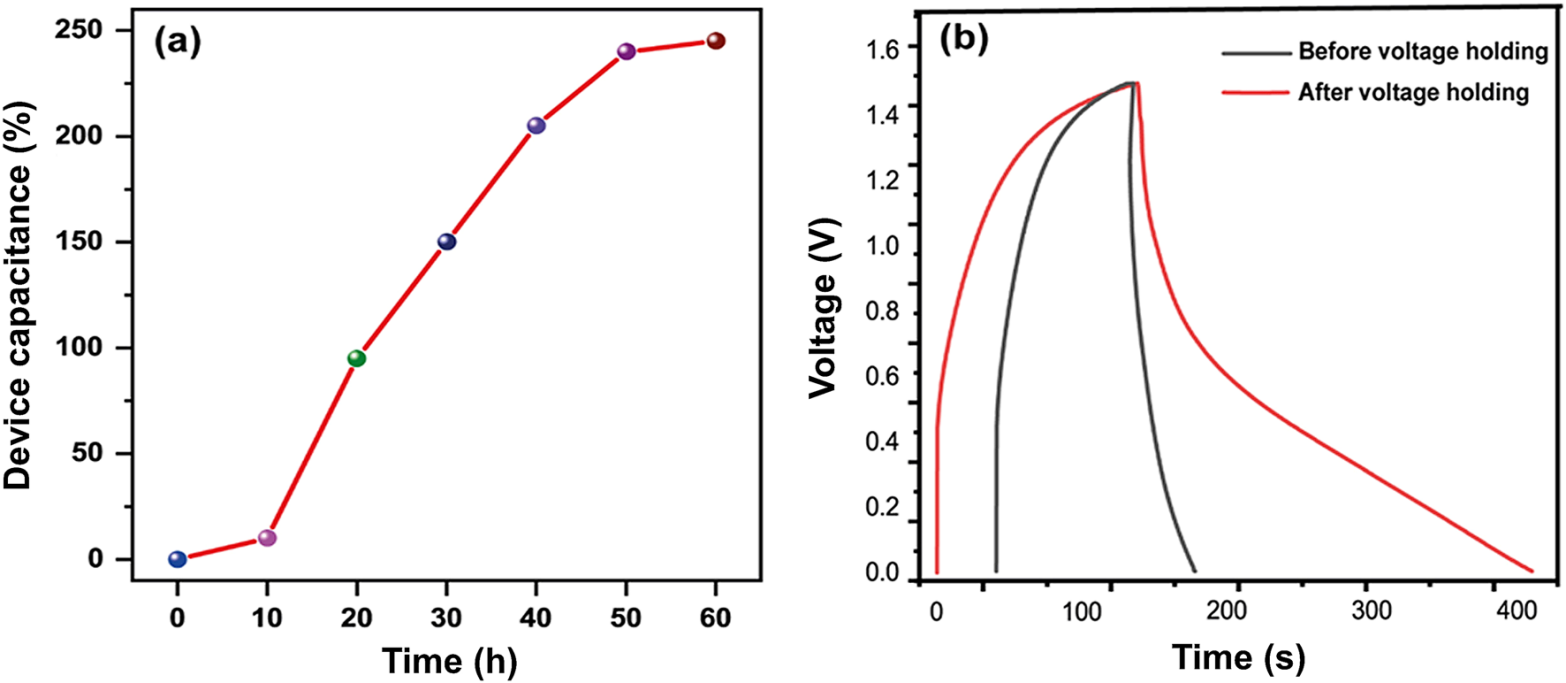
**a** Voltage holding with respect to device capacitance (%) at a current density of 2 A g^−1^. **b** GCD curves of a symmetric device with MnO_x_/C-rGO before and after voltage holding test at 2 A g^−1^ in 1.0 M Na_2_SO_4_ solution

**Fig. 7:**
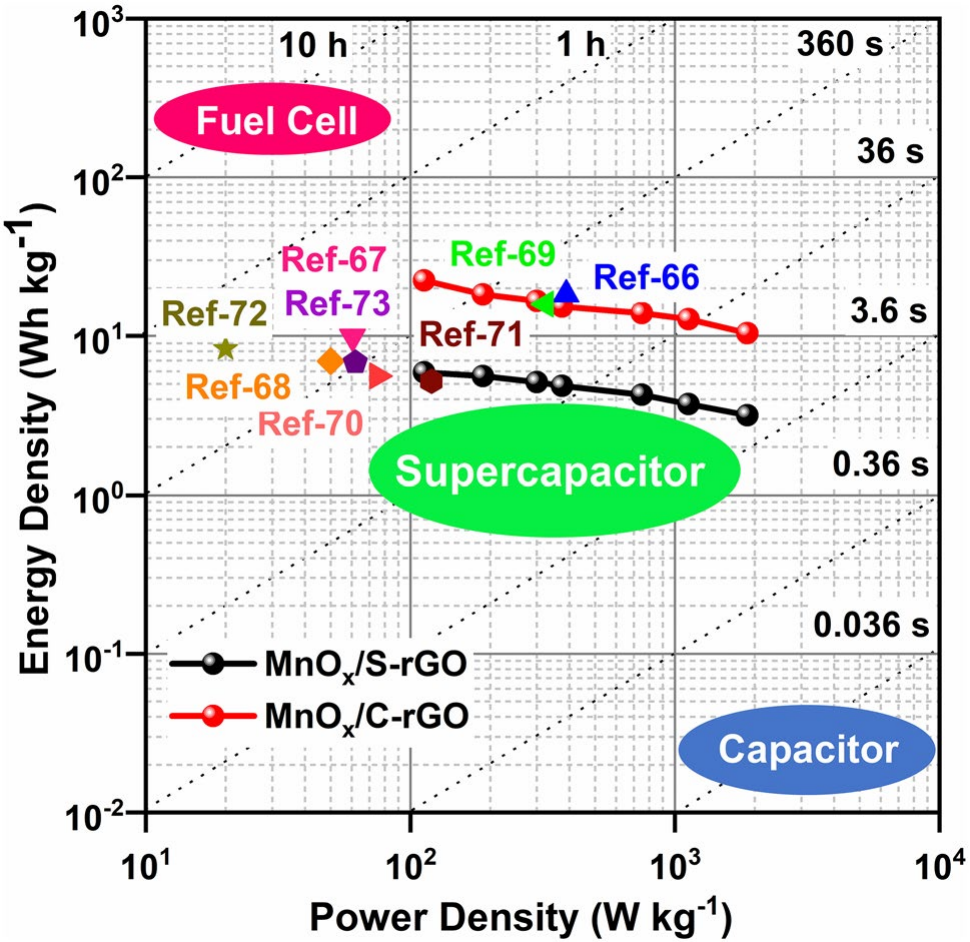
Ragone plots of the symmetric devices with MnO_x_/S-rGO and MnO_x_/C-rGO electrodes

The voltage holding (VH) test of MnO_x_/C-rGO was performed to investigate the reliability of the material upon exposure to the maximum working potential over a considerable time duration. A symmetric device with MnO_x_/C-rGO electrode was subjected to a maximum voltage of 1.5 V for 60 h. Figure 6a displays the variation of *C*_sp_ with each holding time of 10 h. The *C*_sp_ increases with increasing holding time. After 60 h, the *C*_sp_ rises to 250% of its initial capacitance. This may be attributed to the activation of surface charges and pores of MnO_x_/C-rGO during the retention of voltage holding for a certain time duration. This is supported by comparing the GCD curves of the MnO_x_/C-rGO-based device before and after the VH test. An improvement of the discharge time is evident after 60 h holding time (Fig. 6b). We have compared the energy density (*E*) and power density (*P*) of the symmetric devices with MnO_x_/C-rGO and MnO_x_/S-rGO in the Ragone plot diagram, as shown in Fig. 7. The *E* and *P* values were calculated using Eqs. (3) and (4) from the GCD curves. The GCD curves are shown in Additional file 1: Fig. S8c, d. The *E* values of the MnO_x_/C-rGO-based device (23 Wh kg^−1^) are considerably higher than the MnO_x_/S-rGO-based device (5 Wh kg^−1^) at all current densities. The *E* of the supercapacitor depends on the cell voltage and specific capacitance. Understandably, the reason for the high *E* of the MnO_x_/C-rGO-based device lies in its high capacitance values. Due to the presence of crushed graphene, the energy density of a symmetric device with MnO_x_/C-rGO is enhanced without comprising the *P* values. Supercapacitors with high *P* and *E* values are highly desired for modern electronic devices to compete with lithium-ion batteries. Additionally, it is observed that the *E* value of the MnO_x_/C-rGO-based device is higher than those of the devices with various rGO composites reported in the literature (Fig. 7) [66–73].

**Fig. 8:**
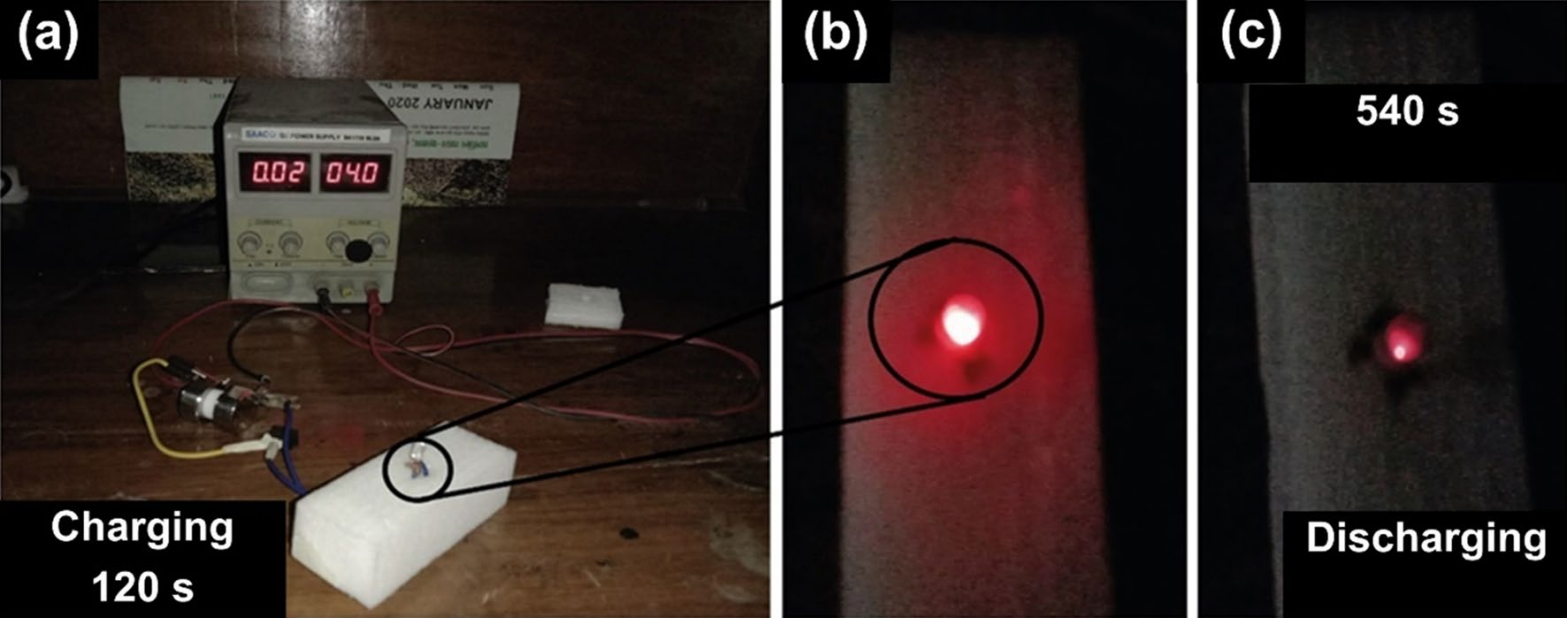
**a** The mini-prototype device of MnO_x_/C-rGO-based symmetric supercapacitor; **b** and **c** response tracking with the red LED light

To demonstrate the practical applicability of the MnO_x_/C-rGO composite, a mini-prototype device was fabricated. The photograph of the fabricated symmetric supercapacitor device is shown in Fig. 8a. The device was charged at 4.0 V using an electrometer for 120 s. During discharging, an LED light was connected to the device to track the response. The assembled supercapacitor can power a red LED light during discharging, as shown in Fig. 8b, c. After charging for 120 s, the device can power the LED light for about 540 s. These results indicate the promising practicality of the MnO_x_/C-rGO-based symmetric device.

## Conclusions

In summary, we have demonstrated a facile route to prepare MnO_x_/C-rGO with high-level 3D structural disorders and exfoliated structures, which show high electrical conductivity and good structural integrity. The agglomeration and stacking problems of graphene sheets have been successfully minimized using a crushed 3D structure. The electrochemical measurements show that MnO_x_/C-rGO exhibits a considerably higher specific capacitance (863 F g^−1^) than MnO_x_/S-rGO (373 F g^−1^) and MnO_x_ (200 F g^−1^) at a current density of 0.2 A g^−1^. When assembled into a symmetric supercapacitor, the MnO_x_/C-rGO-based device exhibits a higher *C*_sp_ (288 F g^−1^) than the MnO_x_/S-rGO-based device (75 F g^−1^) at a current density of 0.3 A g^−1^. Moreover, the MnO_x_/C-rGO-based device exhibits a higher energy density of 23 Wh kg^−1^ than the MnO_x_/S-rGO-based device (5 Wh kg^−1^) at a power density of 113 W kg^−1^, further demonstrating the favorable assembly of chemically interface-tailored MnO_x_/C-rGO for use as a supercapacitor electrode. Such self-supporting 3D architecture avoids the occurrence of “dead mass” caused by the conductive agents and binders during cycling and exhibits excellent electrochemical performance, which will provide a promising candidate for next-generation electrochemical Na-storage systems.

## Supplementary Information

**Additional file 1: Figure S1.** TEM images of (a) MnO_x_/S-rGO and (b) MnO_x_/C-rGO and HRTEM images of (c) MnO_x_/S-rGO and (d) MnO_x_/C-rGO. **Figure S2.** High-resolution O1s XPS spectra of (a) MnO_x_/S-rGO and (b) MnO_x_/C-rGO. **Figure S3.** High resolution C 1 s XPS spectra of (a) MnO_x_/S-rGO and (b) MnO_x_/C-rGO. **Figure S4.** High resolution Mn 2p XPS spectra of (a) MnO_x_/S-rGO and (b) MnO_x_/C-rGO. **Figure S5.** (a) CV curves at different scan rates and (b) GCD curves at different current densities of MnO_x_ in 0.5 M Na_2_SO_4_ electrolyte in the potential range of − 0.1 to 0.8 V. **Figure S6.** Electrochemical study in 0.5 M Na_2_SO_4_ electrolyte with a three-electrode system: (a) CV curves at different scan rates and (b) GCD curves at different current densities of MnO_x_/S-rGO in the potential range of − 0.1 to 0.8 V. **Figure S7.** (a) CV curves at different scan rates and (b) GCD at different current densities of MnO_x_/C-rGO in 0.5 M Na_2_SO_4_ electrolyte in the potential range of − 0.1 to 0.8 V. **Figure S8.** Electrochemical study in 1 M Na_2_SO_4_ electrolyte with a two-electrode system. CV curves of the symmetric devices prepared with (a) MnO_x_/S-rGO and (b) MnO_x_/C-rGO at different scan rates. GCD curves of the symmetric devices prepared with (c) MnO_x_/S-rGO and (d) MnO_x_/C-rGO at different current densities in the voltage range of 0 to 1.5 V. **Figure S9.** Equivalent fitting circuit. **Table. S1** The obtained values of *R*_S_, *R*_ct_, *C*_dl_, *Z*_w_, *C*_p_, from EIS fitting.

## Data Availability

The datasets used and/or analyzed during the current study are available from the corresponding author on reasonable request.
