# Supplementary material for: Boosting capacitive performance of manganese oxide nanorods by decorating with three-dimensional crushed graphene
Source: Nano Converg. 2022 Feb 21;9:10. doi: 10.1186/s40580-022-00300-2 (PMC8861250; doi:10.1186/s40580-022-00300-2)
Supplement: Supplementary file 1 — Additional file 1: Figure S1. TEM images of (a) MnOx/S-rGO and (b) MnOx/C-rGO and HRTEM images of (c) MnOx/S-rGO and (d) MnOx/C-rGO. Figure S2. High-resolution O1s XPS spectra of (a) MnOx/S-rGO and (b) MnOx/C-rGO. Figure S3. High resolution C 1 s XPS spectra of (a) MnOx/S-rGO and (b) MnOx/C-rGO. Figure S4. High resolution Mn 2p XPS spectra of (a) MnOx/S-rGO and (b) MnOx/C-rGO. Figure S5. (a) CV curves at different scan rates and (b) GCD curves at different current densities of MnOx in 0.5 M Na2SO4 electrolyte in the potential range of − 0.1 to 0.8 V. Figure S6. Electrochemical study in 0.5 M Na2SO4 electrolyte with a three-electrode system: (a) CV curves at different scan rates and (b) GCD curves at different current densities of MnOx/S-rGO in the potential range of − 0.1 to 0.8 V. Figure S7. (a) CV curves at different scan rates and (b) GCD at different current densities of MnOx/C-rGO in 0.5 M Na2SO4 electrolyte in the potential range of − 0.1 to 0.8 V. Figure S8. Electrochemical study in 1 M Na2SO4 electrolyte with a two-electrode system. CV curves of the symmetric devices prepared with (a) MnOx/S-rGO and (b) MnOx/C-rGO at different scan rates. GCD curves of the symmetric devices prepared with (c) MnOx/S-rGO and (d) MnOx/C-rGO at different current densities in the voltage range of 0 to 1.5 V. Figure S9. Equivalent fitting circuit. Table. S1 The obtained values of RS, Rct, Cdl, Zw, Cp, from EIS fitting. [file 40580_2022_300_MOESM1_ESM.docx]

***Electronic Supporting Information***

**Boosting Capacitive Performance of Manganese Oxide Nanorods by Decorating with Three-Dimensional Crushed Graphene**

Akter Hossain Reaz^1^, Shimul Saha^2^, Chanchal Kumar Roy^1^, Md Abdul Wahab^3^, Geoffrey Will^4^, Mohammed A Amin^5^, Yusuke Yamauchi^3,6^, Shude Liu^6^*, Yusuf Valentino Kaneti^3^*, Md Shahriar Hossain^7^, and Shakhawat H. Firoz^1^*

*^1^Department of Chemistry, Bangladesh University of Engineering and Technology, Dhaka 1000, Bangladesh.*

*^2^Department of Chemistry, Jashore University of Science and Technology, Jashore 7408, Bangladesh.*

*^3^Australian Institute for Bioengineering and Nanotechnology (AIBN), The University of Queensland, Brisbane, QLD 4072, Australia.*

*^4^School of Mechanical, Medical and Process Engineering, Faculty of Engineering, Queensland University of Technology, Brisbane City, 4000, QLD, Australia.*

*^5^* *Department of Chemistry, College of Science, Taif University, P.O. Box 11099, Taif 21944, Saudi Arabia.*

*^6^JST-ERATO Yamauchi Materials Space-Tectonics Project and International Center for Materials Nanoarchitectonics (WPI-MANA), National Institute for Materials Science, Tsukuba, Ibaraki 305-0044, Japan.*

*^7^School of Mechanical and Mining Engineering, Faculty of Engineering, Architecture, and Information Technology (EAIT), The University of Queensland, Brisbane, QLD 4072, Australia.*

**Corresponding authors:** [lsdyy@yonsei.ac.kr](about:blank) (Shude Liu); v.kaneti@uq.edu.au (Yusuf Valentino Kaneti) shfiroz@chem.buet.ac.bd (Shakhawat H. Firoz)


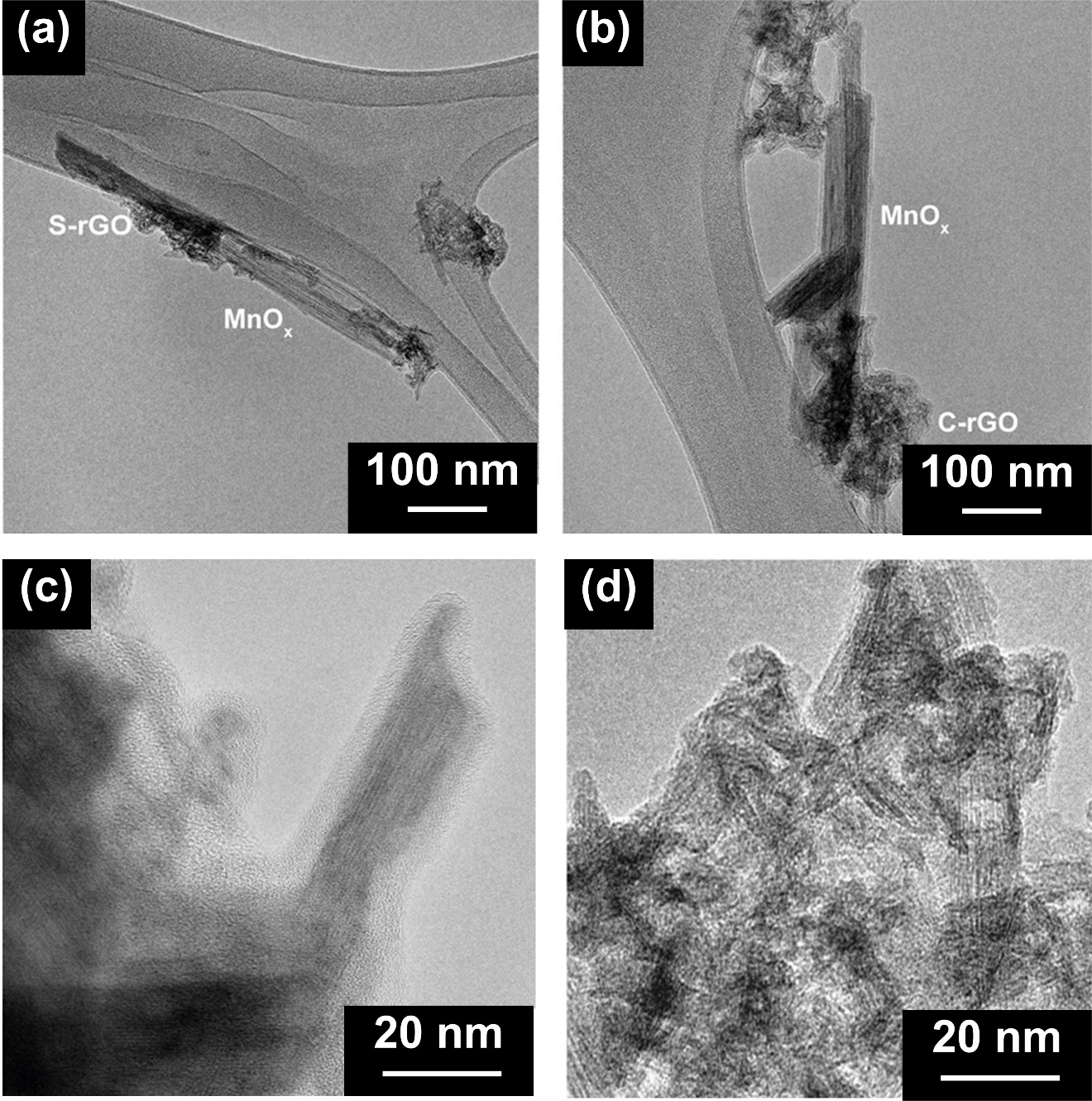


**Figure S1.** TEM images of (a) MnO_x_/S-rGO and (b) MnO_x_/C-rGO and HRTEM images of (c) MnO_x_/S-rGO and (d) MnO_x_/C-rGO.


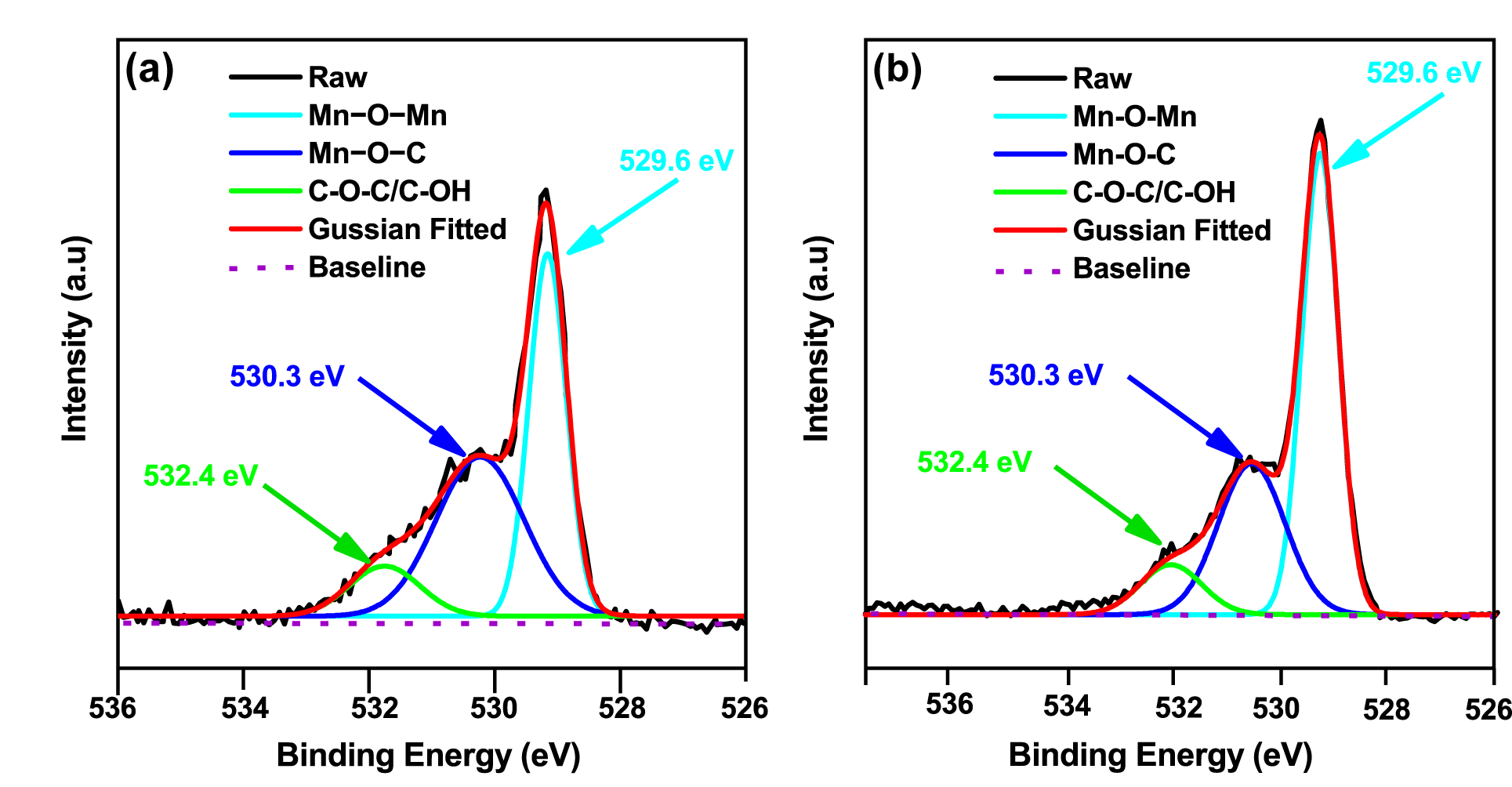


**Figure S2.** High-resolution O1s XPS spectra of (a) MnO_x_/S-rGO and (b) MnO_x_/C-rGO.


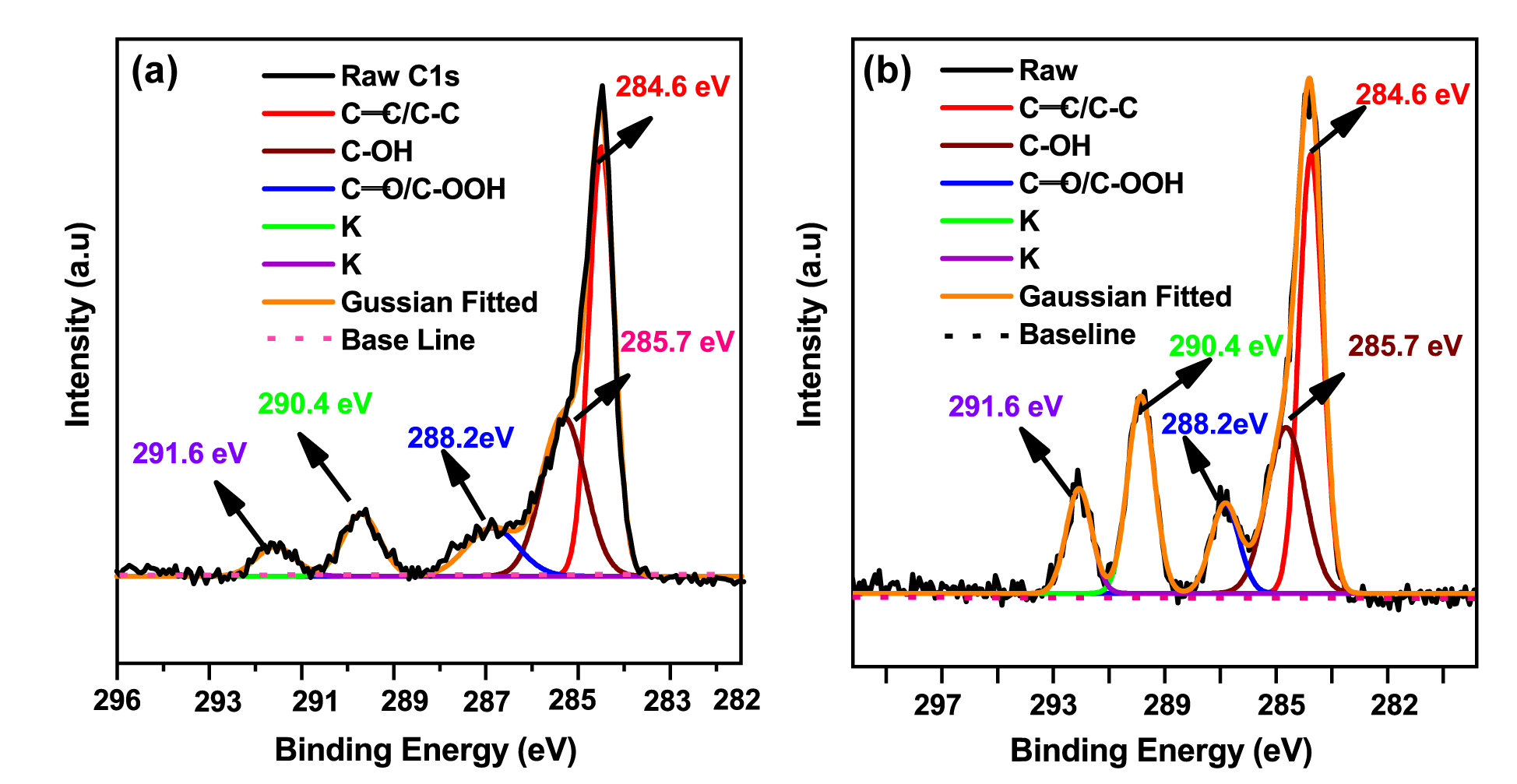


**Figure S3.** High resolution C 1s XPS spectra of (a) MnO_x_/S-rGO and (b) MnO_x_/C-rGO.


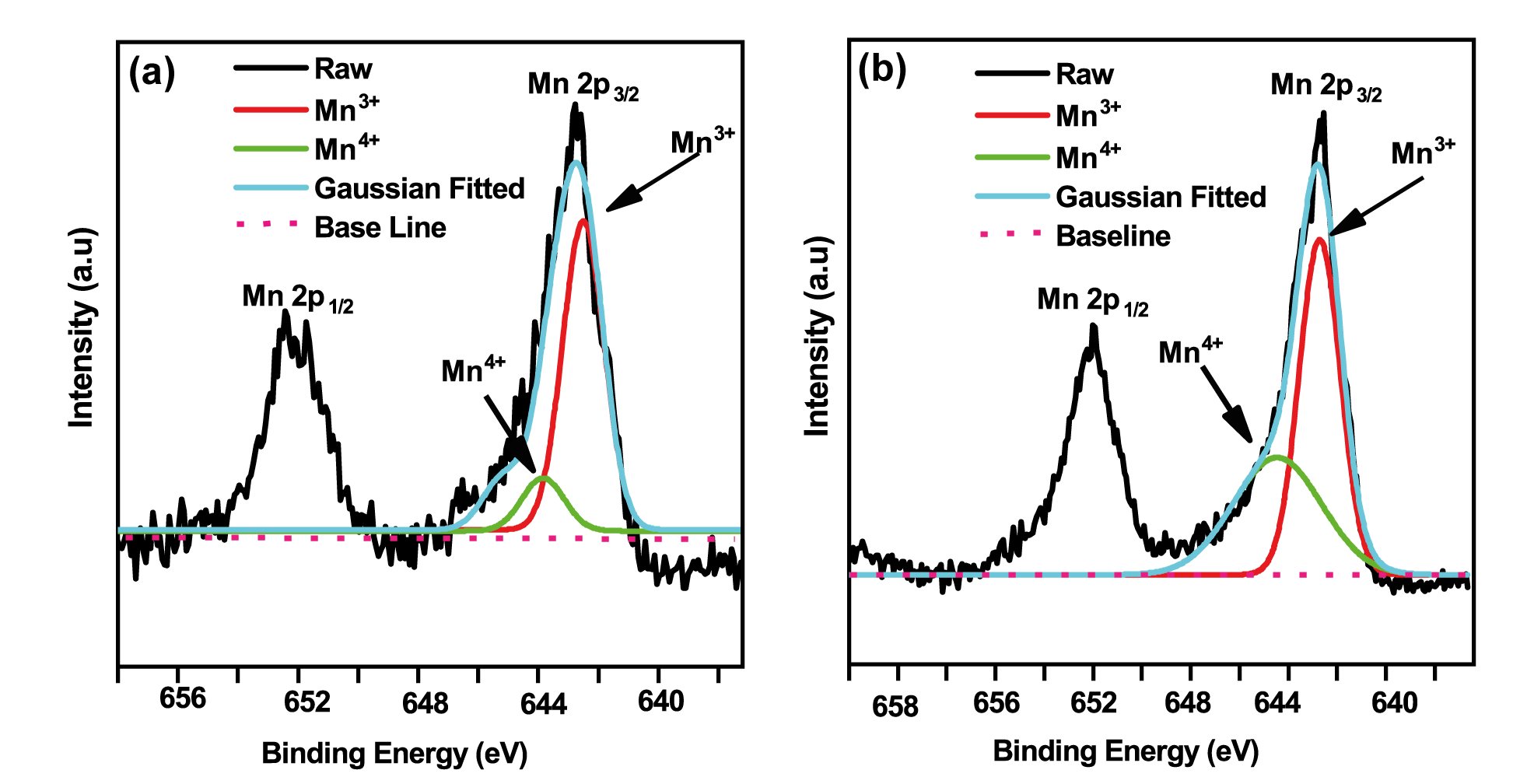


**Figure S4.** High resolution Mn 2p XPS spectra of (a) MnO_x_/S-rGO and (b) MnO_x_/C-rGO.

**
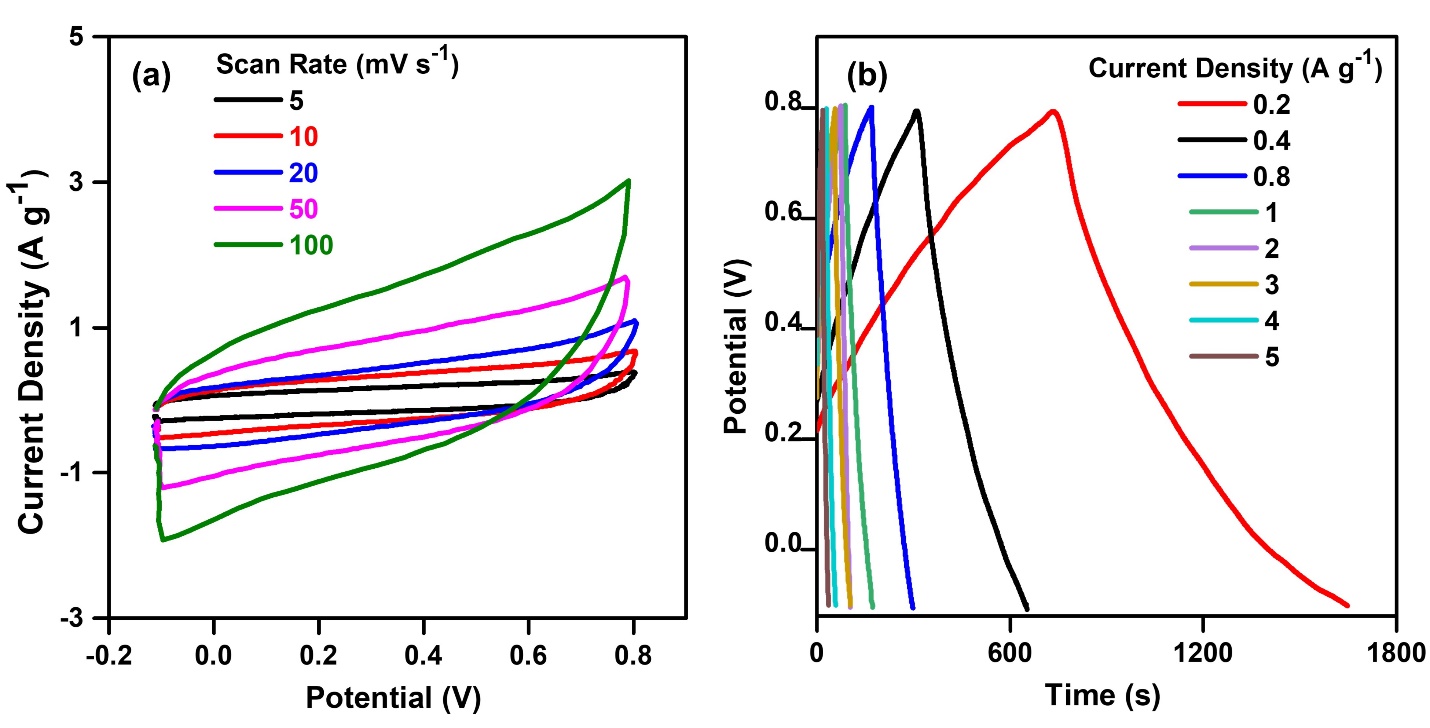
**

**Figure S5.** (a) CV curves at different scan rates and (b) GCD curves at different current densities of MnO_x_ in 0.5 M Na_2_SO_4_ electrolyte in the potential range of −0.1 to 0.8 V.


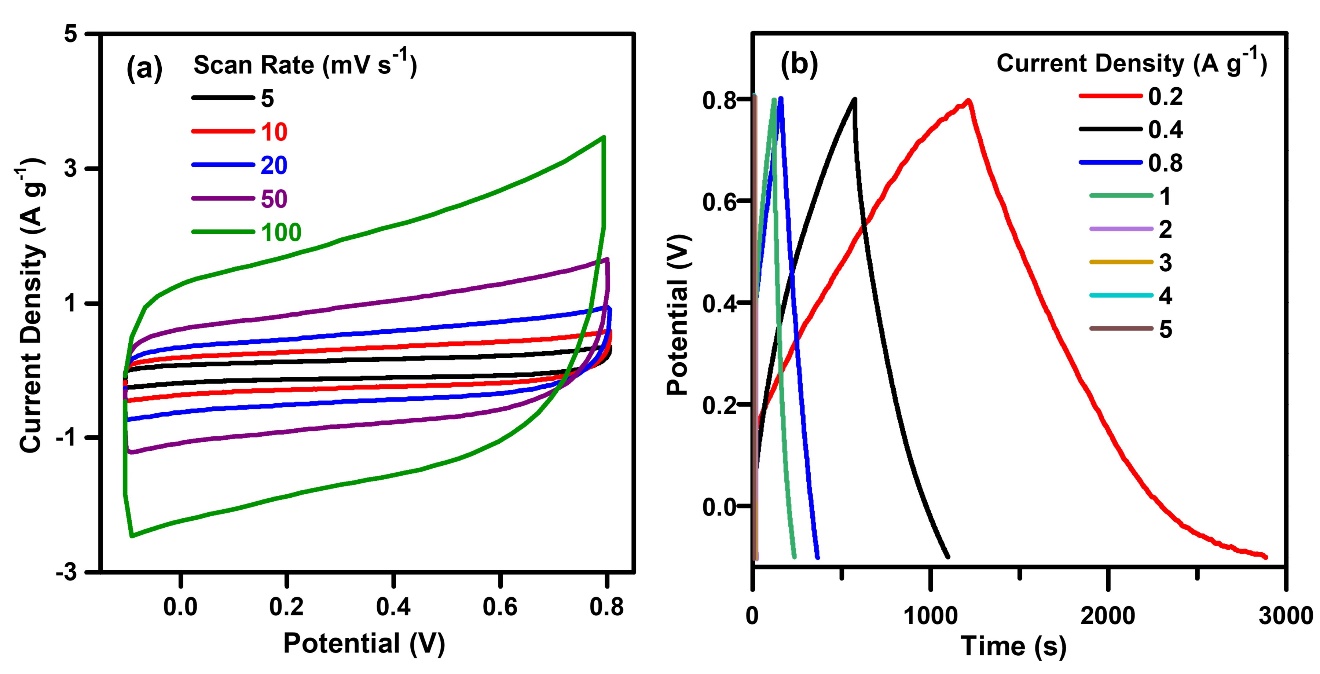


**Figure S6.** Electrochemical study in 0.5 M Na_2_SO_4_ electrolyte with a three-electrode system: (a) CV curves at different scan rates and (b) GCD curves at different current densities of MnO_x_/S-rGO in the potential range of −0.1 to 0.8 V.

.


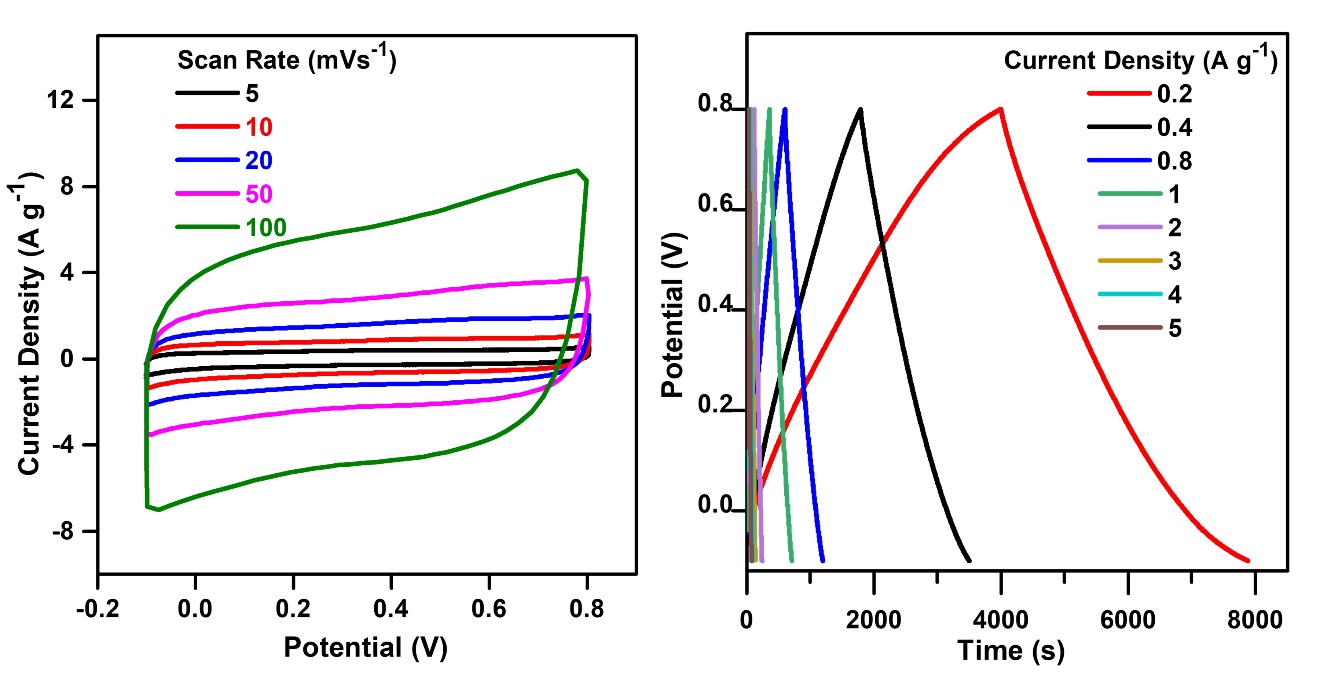


**Figure S7.** (a) CV curves at different scan rates and (b) GCD at different current densities of MnO_x_/C-rGO in 0.5 M Na_2_SO_4_ electrolyte in the potential range of −0.1 to 0.8 V.


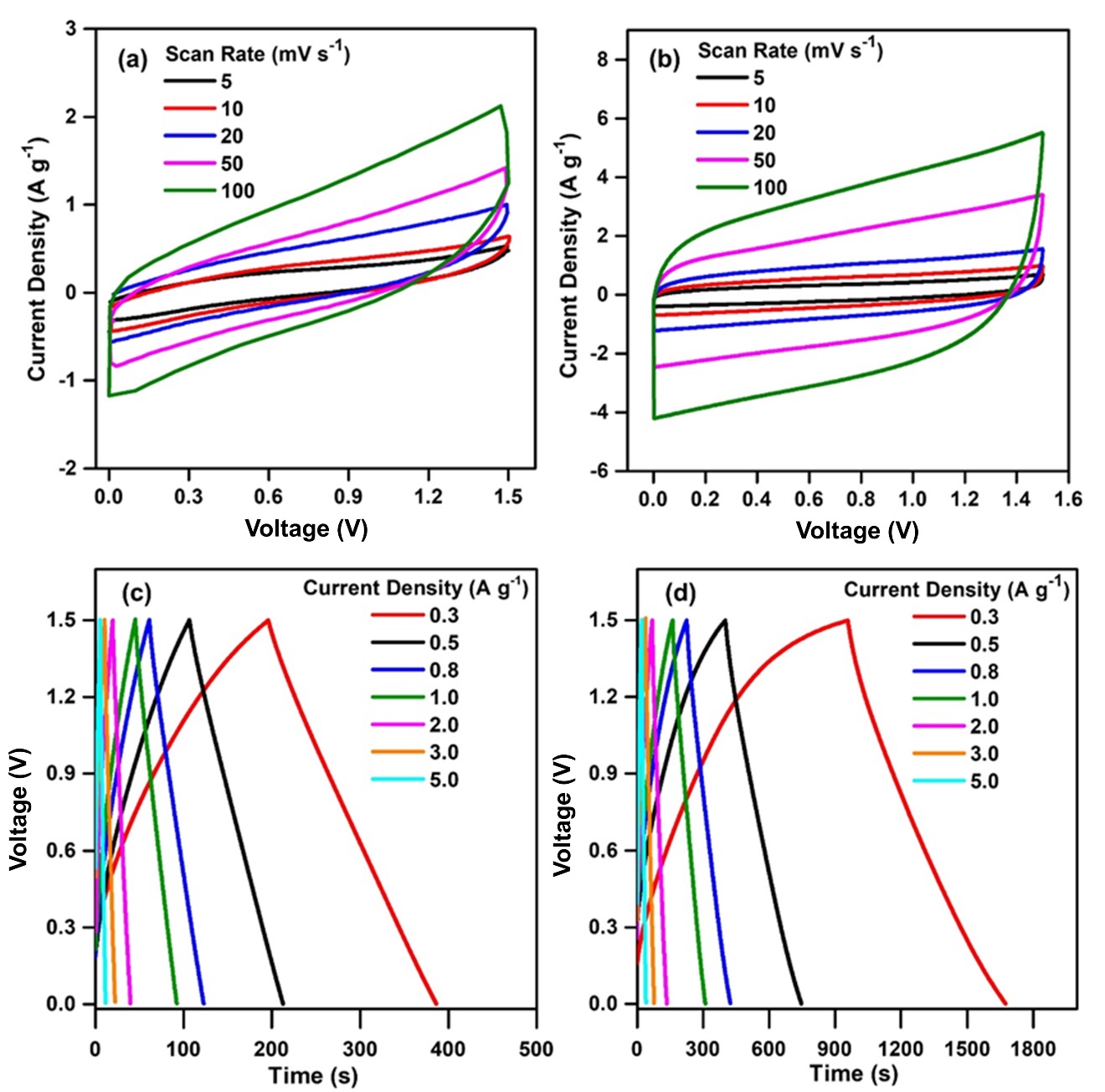


**Figure S8.** Electrochemical study in 1 M Na_2_SO_4_ electrolyte with a two-electrode system. CV curves of the symmetric devices prepared with (a) MnO_x_/S-rGO and (b) MnO_x_/C-rGO at different scan rates. GCD curves of the symmetric devices prepared with (c) MnO_x_/S-rGO and (d) MnO_x_/C-rGO at different current densities in the voltage range of 0 to 1.5 V.


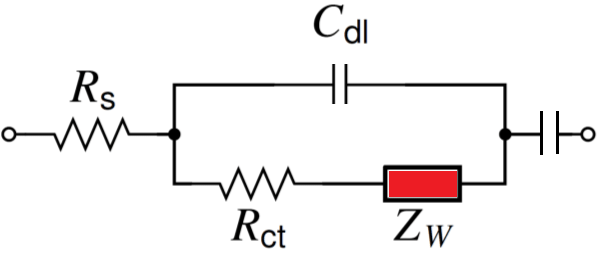


*C*_p_

**Figure S9.** Equivalent fitting circuit

**Table. S1** The obtained values of *R*_S_, *R*_ct_, *C*_dl_, *Z*_w_, *C*_p_, from EIS fitting.

| **Symmetric supercapacitor device** | ***R*_s_ (Ohm)** | ***R*_ct_ (Ohm)** | ***C*_dl_ (µF)** | ***Z*_W_** | ***C*_p_ (F)** |
| --- | --- | --- | --- | --- | --- |
| MnO_x_/S-rGO-based device | 5.531 | 10.454 | 7.21 | 0.01605 | 0.03546 |
| MnO_x_/C-rGO-based device | 3.742 | 8.734 | 4.67 | 0.02194 | 0.03798 |
